# Design and Optimization of a Tapered Magnetic Soft Continuum Robot for Enhanced Navigation in Cerebral Vasculature

**DOI:** 10.3390/mi16060701

**Published:** 2025-06-12

**Authors:** Jiahang Wang, Yuhang Liu, Xiwen Lu, Yunlong Zhu, Chenyao Bai

**Affiliations:** College of Intelligent Robotics and Advanced Manufacturing, Fudan University, 220 Handan Road Yangpu District, Shanghai 200433, China; 23110860061@m.fudan.edu.cn (J.W.); 23210860013@m.fudan.edu.cn (Y.L.); xwlu21@m.fudan.edu.cn (X.L.); zyl@fudan.edu.cn (Y.Z.)

**Keywords:** magnetic soft continuum robot, gray wolf optimizer, tapered MSCR, vascular intervention

## Abstract

Magnetic soft continuum robots (MSCRs) have broad application advantages in vascular intervention; however, current MSCRs still face challenges in navigating the narrower and tortuous structure of the cerebral vasculature. To address this challenge, we propose a tapered MSCR (T-MSCR), which is designed to facilitate smooth navigation through microvascular structures via its miniature tip. Specifically, to optimize its bending ability, we combine the Gray Wolf Optimizer (GWO) with the Euler–Bernoulli beam theory and introduce a Discrete GWO (DGWO) approach to optimize the distribution of magnetic particles within the T-MSCR. We then demonstrate the optimization process of the T-MSCR’s bending ability, comparing and analyzing its deflection angle and deformation characteristics, highlighting its capability to enter microvasculars. Furthermore, we demonstrate the magnetic steering and path selection capabilities of T-MSCR in a two-dimensional vascular model and its navigation performance in real-scale human vascular models. Finally, biocompatibility tests confirm that T-MSCR exhibits no toxicity to human cells, thereby laying a solid foundation for its clinical application. The proposed T-MSCR design and optimization are expected to provide a more efficient and feasible solution for future cerebrovascular interventions.

## 1. Introduction

**Figure 1:**
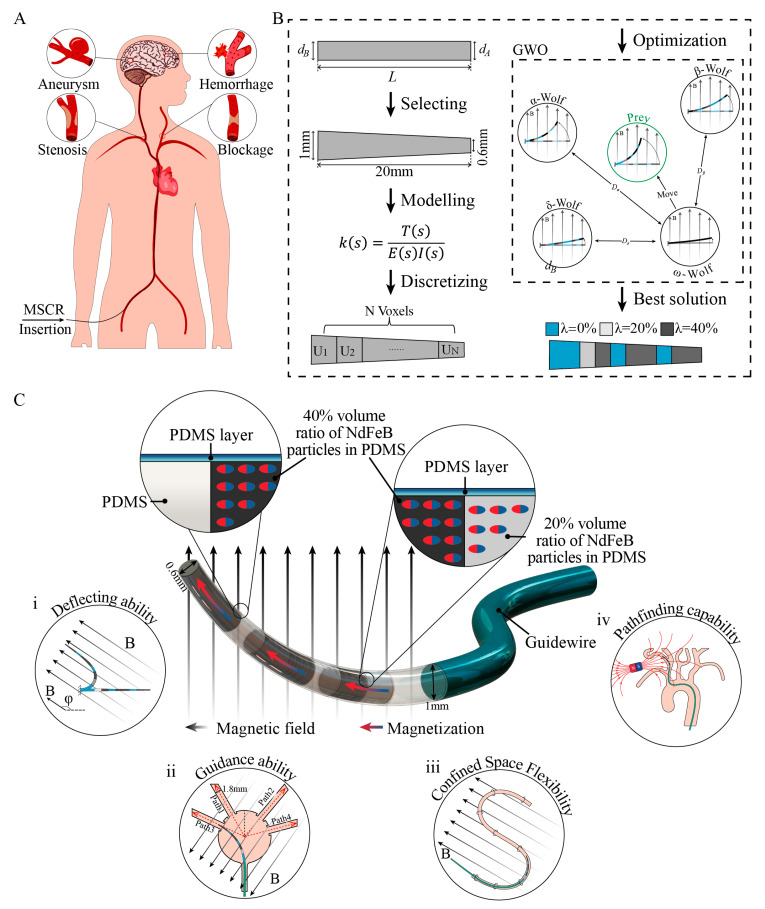
Overview of the design and optimization process of the T-MSCR. (**A**) Cardiovascular diseases can benefit from MSCRs with magnetic navigation capabilities. (**B**) Design and optimization process of T-MSCR. (**C**) Structural illustration of the proposed T-MSCR. This device is affixed to the distal tip of a standard clinical guidewire. It is composed of a composite material incorporating NdFeB magnetic microparticles dispersed in a flexible PDMS base polymer and is encapsulated with a PDMS coating. The designed T-MSCR exhibits good deflection ability, guiding capabilities in narrow channels, magnetic steering, and path selection capabilities in constrained channels, as well as path navigation abilities in three-dimensional blood vessels.

Panvascular diseases are a group of disorders characterized by pathological changes in the vascular system that pose a severe threat to human health [1,2,3]. Globally, more than 20 million people die each year from cardiovascular-related diseases, with cerebral aneurysms, cerebral hemorrhages, and vascular stenosis being the leading causes of death (Figure 1A) [4,5,6]. Cerebrovascular diseases, in particular, are challenging to treat due to the narrow and tortuous structure of the vessels, which significantly complicates precise medical intervention.

Vascular interventional therapy has become the primary treatment for vascular diseases due to its advantages of minimal trauma, rapid recovery, and low cost [7,8,9]. However, traditional interventional procedures rely on X-ray fluoroscopy, which exposes medical personnel to radiation and presents significant operational challenges, demanding high technical proficiency from operators [10,11,12]. In recent years, the development of the remote magnetic-driven guidewire interventional system (MDGIS) has provided a revolutionary solution for vascular interventions [13,14,15]. This MDGIS system connects an MSCR to the front end of a commercial guidewire and uses a magnetic navigation system (MNS) along with a master-slave advancer (MSA) to enable precise remote navigation of the guidewire. The system not only reduces radiation exposure for the operator but also significantly enhances operational accuracy and flexibility.

Although MDGIS has demonstrated significant potential in vascular intervention, challenges remain in achieving flexible navigation for cerebrovascular intervention because of the narrower and more tortuous structure of the cerebral blood vessels [15,16,17]. One limit of its development is the miniaturization of the guidewire. Traditional soft continuum robots often rely on conventional mechanisms such as hydraulic, pneumatic, and cable-driven systems [18,19,20]. These mechanisms are constrained by their inherent structures and drive devices, making it difficult to achieve millimeter- or submillimeter-scale miniaturization. To address this issue, millimeter and submillimeter scale MSCRs have become a focal point of research. Most researchers have integrated tiny permanent magnets into the flexible body of the MSCR, using forces or torques generated by an external magnetic field to control its deformation [21,22,23]. Few studies have explored replacing permanent magnets with micron-sized magnetic particles to overcome the limitations of magnet size and stiffness, thereby further enhancing the miniaturization and flexibility of MSCRs [24,25,26]. However, current approaches still lack sufficient navigational flexibility in the complex cerebral vasculature.

In recent years, programmable magnetization technology has become a key approach for enhancing the flexibility of magnetic particle-embedded MSCRs [25,27,28,29]. By optimizing the distribution of the magnetic particle density and magnetization direction within the MSCR, significant improvements can be made in their navigational performance within complex vascular environments. Current methods for optimizing MSCR designs include physical modeling, deep learning algorithms, and intelligent optimization algorithms [30]. Intelligent optimization algorithms have become one of the most promising approaches because of their theoretically infinite search space and the advantage of not requiring experimental setups based on models. For example, Wu et al. used an evolutionary algorithm to optimize the density and magnetization direction of magnetic particles in each voxel segment with the goal of ideal deformation of a magnetically flexible robot [31]. Wang et al. employed the genetic algorithm to iteratively optimize the magnetic particle distribution of the MSCR by maximizing its physical workspace [32]. Alistair Bacchetti et al. proposed an ideal optimization scheme to optimize the properties of the magnetic particle density, magnetization direction, and stiffness of each voxel segment of the MSCR using an integrated optimization protocol with the objective of target deflection of the MSCR [30]. However, these MSCR optimization methods usually require manual adjustment of numerous control parameters, and the global search capability of the algorithm tends to be low when facing complex optimization problems.

This study proposes a novel T-MSCR, designed to facilitate smooth navigation through microvascular structures via its miniature tip and optimized based on DGWO (Figure 1B). The aim is to enhance its deflection capability by optimizing the distribution of magnetic particles, thereby facilitating the smooth entry of the MSCR into narrow blood vessels (Figure 1C). The fabrication and axial magnetization of the T-MSCR were carried out in accordance with the DGWO optimization results. We demonstrate the optimization process of the T-MSCR’s deflection capability (Figure 1B) and analyze the deflection angle and deformation characteristics through both numerical simulations and comparative experiments. The results show that the T-MSCR exhibits superior deflection ability compared to the same-size cylindrical MSCR with a single-concentration (SC-MSCR), as well as the single-concentration tapered MSCR (ST-MSCR). Furthermore, compared to SC-MSCR, T-MSCR has a stronger guiding ability in narrow channels. The magnetic steering and path selection abilities of the T-MSCR in two-dimensional constrained channels, as well as its navigation ability in realistic-scale human vascular models, are also demonstrated. Finally, biocompatibility tests indicate that T-MSCR was non-toxic to human vascular cells, providing a solid foundation for its clinical applications.

## 2. Materials and Methods

### 2.1. Preparation of Magnetic Composite Material

The magnetic composite was prepared by blending hard magnetic NdFeB microparticles (MQFP-B-2007609-089, Magnequench, Tübingen, Germany) with a flexible polydimethylsiloxane (PDMS) base polymer (SYLGARD 184, Dow Corning, Midland, MI, USA). We define λ as the volume ratio of magnetic particles to PDMS. Based on predetermined λ={0%, 5%, 10%, 15%, 20%, 25%, 30%, 35%, 40%}, NdFeB particles were mixed with the PDMS base resin, and the resulting mixtures were placed into beakers. Subsequently, a curing agent was added to the mixture at a mass ratio of 19:1 (base to curing agent). The resulting formulation was stirred using a mechanical stirrer for 120 s (1000 r/min).

Following mixing, the composite material was transferred to a vacuum drying oven (DZF-6050, Supo Instruments Co., Ltd., Shaoxing, China) and degassed under vacuum conditions for 20 min to eliminate trapped air bubbles. The air-free composite was then poured into prefabricated molds. These molds were subsequently cured in the same vacuum oven (100 °C, 2 h). After curing and demolding, the samples were subjected to axial magnetization using a pulsed magnetizer, which was manufactured by Shengjiayin Electronic Equipment Co., Ltd., Shenzhen, China.

### 2.2. Magnetic Characterization

Rectangular sheets of PDMS and NdFeB composite material (length 70 mm, width 70 mm, height 2 mm) were fabricated via injection molding with λ={5%, 10%, 15%, 20%, 25%, 30%, 35%, 40%}. From these sheets, cubic specimens (2 mm × 2 mm × 2 mm) were precisely sectioned for characterization. Magnetic hysteresis loops were measured by a magnetic measurement system (SQUID-VSM, Quantum Design, San Diego, CA, USA). Then, the density of each specimen, corresponding to its specific λ value, was measured by an electronic density measurement balance. Based on the measured density values, the residual magnetization M of each sample was computed.

### 2.3. Mechanical Testing

Dumbbell-shaped specimens (50 mm in length, 4 mm in width, and 16 mm in gauge length) were prepared from PDMS–NdFeB magnetic composites with varying λ by mold injection and subsequent cutting. Tensile tests were performed using an Instron 5966 universal testing machine (Instron, Norwood, MA, USA) at a loading rate of 10 mm/min. Axial stress–strain curves were recorded, and Young’s modulus was calculated for each specimen.

### 2.4. Blood Vessel Model

The two-dimensional blood vessel model was modeled using computer-aided design software (SolidWorks, Dassault Systemes, Waltham, MA, USA) and fabricated on a 3D printer (MAGE S, Creality 3D Technology Co., Ltd., Shenzhen, China) using photosensitive resin. Both the sodium fatty alcohol sulfate and three-dimensional vascular model (Preclinic Medtech Co., Ltd., Shanghai, China) were used in this study.

### 2.5. Three-Dimensional Helmholtz Coils

In our experiments, we used our previously constructed three-dimensional Helmholtz coil to generate a uniform magnetic field [33], which is a magnetic field system that can generate a uniform magnetic field with a magnetic field strength ranging from 0 to 15 mT in any direction in a spherical space with a diameter of 50 mm.

### 2.6. Magnetic Actuation Model for Permanent Magnets

Among the methods for describing the magnetic field of permanent magnets, the dipole model is often selected due to its computational simplicity. In our experiments, the permanent magnet at the end of the robot arm can be approximated as a point source and therefore it can be reduced to a magnetic dipole model in a non-uniform field. The magnetic field B generated by a magnetic dipole source on the magnetic segment of the T-MSCR can be expressed as follows:

(1) $$B=\frac{\mu_{0}}{4\pi r^{3}}\left[3\left(m\cdot \hat{r}\right)\hat{r}-m\right]$$

where $\mu_{0}=4\pi \times 10^{-7}T\cdot m/A$ is the vacuum permeability, r representing the distance from the magnetic dipole to the point where the magnetic field is computed, $\hat{r}$ is the unit vector pointing from the magnetic dipole to the point where the magnetic field is computed, and m is the magnetic dipole moment of the permanent magnet.

The external magnetic field can exert a magnetic force $F_{m}$ and a magnetic torque $T_{m}$ on the T-MSCR, which together deflect it, both of which can be expressed as follows:

(2) $$F_{m}=\left(m\cdot \nabla \right)B$$

(3) $$T_{m}=m\times B$$

where m is the magnetic moment of the T-MSCR.

### 2.7. Cell Viability Assessment Method

Human umbilical vein endothelial cells (HUVEC) (product no. iCell-h110, Servicebio Technology Co., Ltd., Wuhan, China) frozen in liquid nitrogen were quickly thawed in a 37 °C water bath. The thawed cells were then added to 10 mL of preheated HUVEC-specific medium containing fetal bovine serum (product no. iCell-h110-001b, Servicebio Technology Co., Ltd., Wuhan, China). The mixture was centrifuged at 1000 rpm for 5 min, and the supernatant was discarded. Fresh HUVEC-specific medium containing fetal bovine serum was added to resuspend the cells, which were then transferred to a culture flask (FORMA STERI-CYCLE i 160, ThermoFisher, Shanghai, China) and incubated at 37 °C with 5% CO_2_. Cells growing normally in the culture flask were collected via trypsin digestion and counted using a hemocytometer to determine cell concentration. Based on this, the cells were seeded into a 48-well cell culture plate (product no. 748001, NEST Biotechnology Co., Ltd., Wuxi, China) at an appropriate density, adding 250 μL of cell suspension per well. A sample was added to each experimental well, while the blank control wells were left untreated. Each group (three replicates) and the plates were incubated at 37 °C with 5% CO_2_. After 24, 48, and 72 h of incubation, the morphology of the HUVECs was observed using an inverted fluorescence microscope (CKX53, OLYMPUS, Tokyo, Japan). Subsequently, 25 μL of CCK-8 solution (product no. K101812133EF5E, APExBio, Houston, TX, USA) was added to each well, and the culture plate was returned to the incubator at 37 °C with 5% CO_2_ for an additional 2 h. After incubation, 100 μL from each well was transferred to a 96-well plate (product no. 701001, NEST Biotechnology Co., Ltd., Wuxi, China), and the absorbance at 450 nm was measured using a microplate reader (Feyond-A300, ALLSHENG, Hangzhou, China). Finally, the cell viability was calculated based on the absorbance readings of the experimental, blank, and control wells.

## 3. Results

### 3.1. Hard Magnetic Elastic Line Theory and the Finite Difference Method

To design a T-MSCR suitable for complex cerebrovascular navigation, we modeled its elastic bending behavior under an external magnetic field using the Euler–Bernoulli beam theory and solved it numerically using the finite difference method to predict its deflection angle.

First, the proximal diameter, distal diameter, and length of the T-MSCR are defined as $d_{B}$, $d_{A}$, and L, respectively. The diameter ratio is defined as $\alpha =d_{B}/d_{A}\;(\alpha >1)$. Then, based on the hard magnetic elastic theory [34,35], an analytical model was established for the deformation behavior of the ST-MSCR under a uniform magnetic field using the Euler–Bernoulli beam theory:

(4) $$\mathrm{EI}\frac{d\theta }{ds}=\frac{\pi d_{A}^{2}}{4L^{2}}\int_{s_{0}}^{L}MB\sin(\varphi -\theta (s))Q(s)ds$$

where E, I, s, dθ/ds, $d_{A}$, L, $s_{0}$, φ, B, M, $\theta \left(s\right)$, and Q(s) represent Young’s modulus, cross-sectional moment of inertia, the arc length measured along the elastic line from the origin to any point P (Figure S7), the curvature of the elastic line evaluated at any point P, the distal diameter, the length, the position of the current analysis point, the angle between the external magnetic field direction and the initial direction, the external magnetic field strength, the magnetization strength, the angle at point P relative to the reference direction, and the volume correction term (see the Supplementary Materials) of the ST-MSCR, respectively.

This model describes the deformation behavior of the ST-MSCR under an external magnetic field, and by integration, the deflection angle θ at any position can be calculated. However, due to the complexity of the model, it is difficult to obtain an analytical solution directly. Therefore, for T-MSCRs with different λ, we can apply the finite difference method to solve Equation (4). In this approach, the T-MSCR is divided into N equal-length segments, and the curvature can be approximately expressed as $\kappa (s)=d\theta /ds\approx (\theta_{i}-\theta_{i-1})/\Delta s$, where Δs=L/N and $\theta_{i}$ represents the angle between the reference direction and the i-th segment. Thus, Equation (4) can be rewritten as follows:

(5) $$E_{i}I_{i}\frac{\theta_{i}-\theta_{i-1}}{\Delta s}=\frac{\pi d_{A}^{2}}{4L^{2}}\sum_{q=1}^{N}M_{q}B\sin(\varphi -\theta_{q})Q_{q}\Delta s,i=1,2,3,\ldots ,N$$

Equation (5) represents an N-dimensional nonlinear system of equations. Given the initial value $\theta_{0}=0$, the deflection angle $\theta_{i}$ of each element can be calculated through iterative solving, ultimately yielding the deflection angle $\theta_{N}$ of the T-MSCR (see the Supplementary Materials).

### 3.2. Materials and Structure of T-MSCR

**Figure 2:**
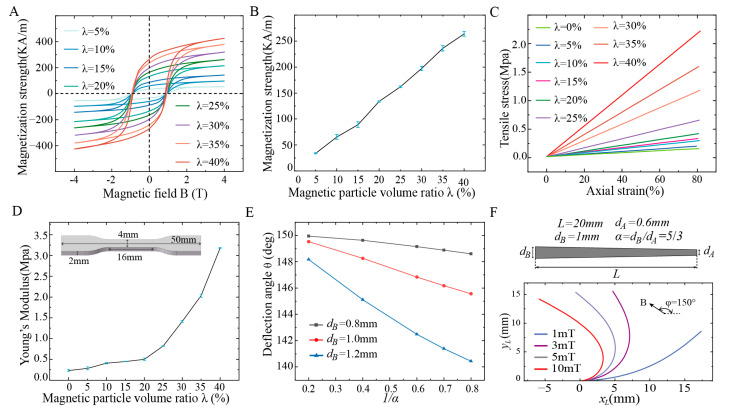
Material characterization and structure of T-MSCR. (**A**) Hysteresis loops of magnetic composite materials with different λ. (**B**) Linear variation of the residual magnetization strength of the composite materials with different λ. (**C**) Stress–strain curves of the magnetic composite materials with increasing λ. (**D**) Young’s modulus of composite materials with increasing λ. (**E**) Numerical simulation of the effect of diameter ratio α on the deflection angle of the ST-MSCR (λ=20%, L=20 mm) under B=10 mT and φ=150°. (**F**) Geometry and deformation of the ST-MSCR with the selected configuration.

The T-MSCR is made from a composite material consisting of hard magnetic NdFeB microparticles and PDMS. The NdFeB particles provide a strong magnetic response capability to the T-MSCR due to their high remanence and coercivity, while PDMS imparts good mechanical deformation properties to the T-MSCR due to its low elastic modulus and high flexibility [36]. To optimize the distribution of λ in the T-MSCR, we prepared test samples with a mass ratio of PDMS substrate material to crosslinking agent of 19:1. First, the density ρ (Figure S5) and magnetic hysteresis loops (Figure 2A) of the samples at λ={5%,10%,15%,20%,25%,30%,35%,40%} were measured using a densimeter and a magnetic measurement system, respectively. The magnetization strength M of the materials was calculated from the hysteresis loops (Figure 2B). Finally, the stress–strain curves (Video S1) and Young’s modulus E of the samples at λ={0%,5%,10%,15%,20%,25%,30%,35%, 40%} were measured using a universal material testing machine (Figure 2C,D). The results showed that λ of the T-MSCR affected both its magnetic response and mechanical flexibility. When λ=40%, the sample exhibited a high magnetization strength (M=263.89 KA/m) as well as a high Young’s modulus (E=3.18 MPa). When λ=0%, the sample had the lowest Young’s modulus (E=0.23 MPa) but the weakest magnetization strength.

The tapered tip of the T-MSCR is specifically designed to facilitate smooth navigation through microvascular structures via its miniature tip. To determine the appropriate *α* of the T-MSCR, numerical simulations were conducted to investigate the effect of α on the deflection angle of the ST-MSCR (λ=20%) with a fixed length of L=20 mm under a uniform magnetic field of B=10 mT (φ=150°), as shown in Figure 2E. The simulation results indicate that increasing the diameter ratio enhances the deflection capability of the ST-MSCR. However, overly small diameters of the T-MSCR tip pose significant fabrication challenges.

Taking into account simulation results, practical considerations, and the typical diameters of cerebral blood vessels at common sites of cerebral aneurysms [37,38,39], hemorrhages, and vascular stenosis, a T-MSCR configuration was selected with a diameter ratio of α=5⁄3, a distal diameter of $d_{A}=0.6\;mm$, a proximal diameter of $d_{B}=1\;mm$, a total length of L=20 mm for experimental validation (Figure 2F). Figure 2F shows the corresponding deformation of the ST-MSCR (λ=20%) when exposed to a uniform magnetic field of B=10 mT (φ=150°) for this configuration. For applications requiring access to smaller microvascular structures, configurations with smaller proximal diameters and diameter ratios may be employed as needed. Additionally, to enhance the surface quality of the T-MSCR and prevent potential harm from exposed magnetic particles to the human body [40,41], the surface was coated with a layer of PDMS.

### 3.3. Optimization of the T-MSCR

**Figure 3:**
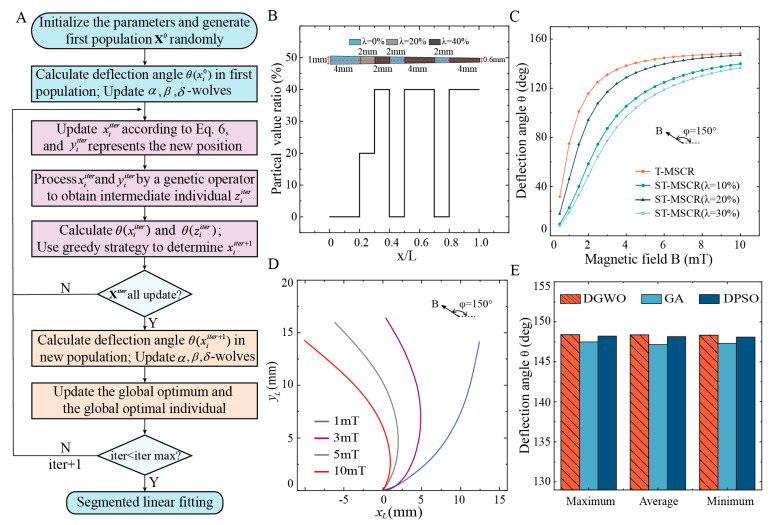
Optimization of T-MSCR. (**A**) Diagram illustrating the optimization process for the distribution of λ in T-MSCR based on DGWO. (**B**) Optimal magnetic particle distribution of T-MSCR after 123 iterations of optimization. (**C**) Theoretical deflection angle comparison between T-MSCR and ST-MSCR at λ={10%, 20%, 30%}. (**D**) Comparison of the theoretical deformation of T-MSCR under $B=\left\{1,3,5,10\;mT\right\}$ and φ=150°. (**E**) Maximum, average, and minimum deflection angles achieved by DGWO, GA, and DPSO across 10 independent optimization attempts for the T-MSCR.

As λ increases, the concentration of magnetic particles within the T-MSCR rises, enhancing the magnetic torque generated under the same magnetic field. Nevertheless, this increase also elevates the bending stiffness, which counteracts the gain in bending performance due to the competing nature of these two effects. Therefore, to improve the bending performance of the T-MSCR, the distribution of λ  was optimized by DGWO, as illustrated in Figure 3A.

The GWO mimics the hierarchical structure of gray wolves (alpha, beta, delta, and omega) and simulates their cooperative hunting behavior. Position updates are guided by leadership dominance and collective behaviors such as tracking and encircling, which drive the algorithm toward the optimal solution [42]. Relative to other intelligent optimization approaches, GWO offers simplicity with fewer control parameters and robust global exploration capabilities, enabling its application across diverse domains [43,44]. However, the GWO is specifically designed for continuous domains, making it unsuitable for direct application to discrete problems. To address this, we propose a discrete GWO (DGWO), which introduces a modulo-based constraint to limit candidate values of λ and integrates a uniform crossover strategy to enhance exploration. With these improvements, the DGWO can more efficiently optimize the distribution of λ.

As illustrated in Figure 3A, the DGWO begins by segmenting the T-MSCR into 100 uniformly sized voxels. Each voxel is randomly assigned an integer value q ∈ {0, 1, 2, …, 8} corresponding to a magnetic particle volume ratio (for example, q=0 represents $\lambda_{i}=0\%$, q=1 represents $\lambda_{i}=5\%$, …, q=8 represents $\lambda_{i}=40\%$). We first generate an initial population $X^{0}$ consisting of 100 individuals, then set an external magnetic field with B=10 mT and φ=150°. For each individual, the deflection angle $\theta (x_{i}^{0})$ is calculated. Alpha, beta, and delta wolves correspond to the three individuals with the largest $\theta (x_{i}^{0})$, respectively. The modulo operation was introduced to control the range of λ. By updating the positions of the alpha, beta, and delta gray wolves in the population, the algorithm approaches the optimal solution.

(6) $$y_{i}^{iter}=\left(x_{\alpha }^{iter}+x_{\beta }^{iter}+x_{\delta }^{iter}\right)\mathrm{mod}\;v$$

where v is the number of discrete values for $\lambda_{i}$ (v=9); $x_{\alpha }^{iter}$, $x_{\beta }^{iter}$, and $x_{\delta }^{iter}$ denote the position of the alpha, beta, and delta wolves at the iter-th iteration, respectively; $y_{i}^{iter}$ represents the updated position of the gray wolves.

To improve the global search capability, we adopted a genetic operator. In particular, the old position $x_{i}^{iter}$ and the new position $y_{i}^{iter}$ of the gray wolf i were paired, and a portion of their voxels was randomly exchanged to create an intermediate position $z_{i}^{iter}$. Then, $\theta (y_{i}^{iter})$ and $\theta (z_{i}^{iter})$ were calculated. A greedy approach was applied to determine the position $x_{i}^{iter+1}$ for gray wolf i in the next iteration (Equation (7)). After completing the position updates within the previous population, we calculated $\theta (x_{i}^{iter+1})$ for each offspring and updated the alpha, beta, and delta wolves accordingly. To ensure the optimal solution is retained and to enhance convergence efficiency, we tracked the best historical values of θ and the corresponding optimal individual (magnetic particle distribution) throughout the iterations. These steps were repeated until the algorithm reached its maximum iteration limit.

(7) $$x_{i}^{iter+1}=\left\{\begin{array}{ll} y_{i}^{iter}, & \theta (y_{i}^{iter})>\theta \left(z_{i}^{iter}\right)\\ z_{i}^{iter}, & otherwise \end{array}\right.$$

Figure S6 demonstrates the iterative optimization process of the deflection angle of the T-MSCR using the DGWO. The maximum deflection angle increased progressively with each iteration and reached a convergent value of 148.38° at the 123rd iteration. Considering the convenience of experimental fabrication, we further performed a segmented linear fitting of the optimized magnetic particle distribution of the T-MSCR (see the Supplementary Materials), and the fitted distribution is shown in Figure 3B. The DGWO-optimized T-MSCR was divided into seven segments, with λ and number of voxels n in each segment being λ={0%,20%,40%,0%,40%,0%,40%} and n={20,10,10,10,20,10,20}, respectively. Moreover, we investigated the influence of the magnetic field orientation angle φ on the segmented optimization results of the T-MSCR (as shown in Figure S6). The results indicated that φ had no significant effect on the optimized configuration. Deflection angles for both the T-MSCR and ST-MSCRs (λ={10%, 20%, 30%}) were analyzed through the finite difference model. Subsequently, we visualized the deformation behavior of the T-MSCR under B={1, 3, 5, 10 mT} at φ=150°, as illustrated in Figure 3C,D. The comparison indicates that the optimized particle distribution markedly improves the overall magnetic response bending performance of the T-MSCR.

To evaluate the global search capability of the DGWO, we compared its performance (without segmented linear fitting) with that of the Genetic Algorithm (GA) and Discrete Particle Swarm Optimization (DPSO) in maximizing the deflection angle of the T-MSCR. Detailed implementations of GA and DPSO are provided in the Supplementary Materials. To obtain statistically reliable results, each algorithm was independently run ten times. DGWO achieved a maximum deflection angle of 148.36 ± 0.03° (mean ± SD), whereas GA and DPSO reached 147.30 ± 0.15° and 148.16 ± 0.06°, respectively. Figure 3E presents the maximum, average, and minimum deflection angles from multiple runs of each algorithm. These results demonstrate that DGWO outperforms GA and DPSO in terms of global search capability for deflection angle optimization. Moreover, DGWO requires fewer tunable parameters, reducing sensitivity to parameter settings compared to GA and DPSO.

### 3.4. Fabrication and Properties of T-MSCR

**Figure 4:**
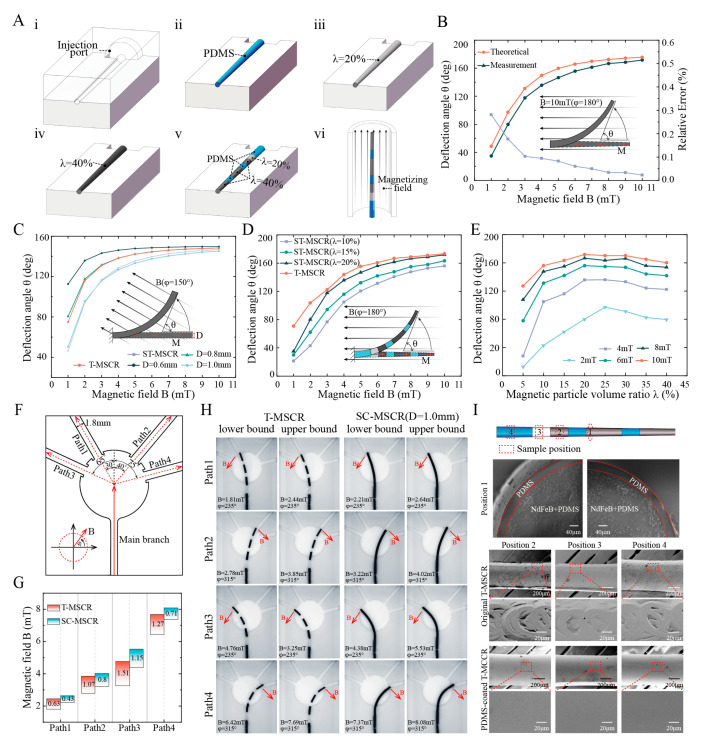
Fabrication and Properties of T-MSCR. (**A**) Schematic illustration of the fabrication process: (i) Machined mold with an injection port based on the T-MSCR geometry; (ii–iv) Preparation of ST-MSCRs by injecting magnetic composite material with *λ* = 0%, 20%, and 40%, respectively; (v) Assembly of the T-MSCR by bonding sub-segments with different *λ* values (0%, 20%, and 40%) using PDMS; (vi) Magnetization of the assembled T-MSCR in an axial magnetic field. (**B**) Comparison between the theoretical and measured deflection angles of ST-MSCR (λ=20%) at various magnetic field strengths. (**C**) Theoretical deflection angle comparison of T-MSCR, ST-MSCR (λ=20%), and SC-MSCR (λ=20%) at various magnetic field strengths. (**D**) Measured deflection angle comparison of T-MSCR and ST-MSCR at various magnetic field strengths. (**E**) Influence of λ on the deflection angle of ST-MSCR at various magnetic field strengths (φ=180°). (**F**) Path model with four branches having different turning angles. (**G**) Comparison of the magnetic field strength range for T-MSCR and SC-MSCR (D = 1.0 mm, λ = 20%). (**H**) Upper and lower bounds of the magnetic field strength and direction for T-MSCR and SC-MSCR passing through the four-branch path. (**I**) Cross-sectional and surface SEM images of T-MSCR, showing a PDMS coating applied to the surface for improved biocompatibility.

Figure 4A illustrates the fabrication process of the T-MSCR. First, we fabricated a mold according to the dimensions of the T-MSCR using machining, as shown in Figure 4A(i). Then, we used the mold injection molding method to prepare ST-MSCRs with λ={0%, 20%, 40%}, as shown in Figure 4A(ii–iv). Next, based on the DGWO optimization results, we extracted the sub-segments corresponding to these three ST-MSCRs and bonded them using PDMS, as shown in Figure 4A(v). Additionally, to improve the surface quality and biocompatibility of the T-MSCR, we coated the surface of the T-MSCR with a layer of PDMS. After curing, the T-MSCR was subjected to axial magnetization using a pulsed magnetizer. Finally, the fabricated T-MSCR was integrated with the distal end of a commercial guidewire (length: 260 cm; diameter: 0.89 mm), enabling its application in vascular navigation procedures. Further details of the T-MSCR fabrication process are provided in Supplementary Materials Note S7.

We fabricated ST-MSCR with λ=20% and compared the theoretical model with the actual measured deflection angle θ of the ST-MSCR under magnetic field strengths ranging from 1 to 10 mT (φ=180°) (Figure 4B). The results demonstrated that the theoretical and measured values exhibited consistent trends in deflection angle variation. This consistency can be attributed to the fact that, as the applied magnetic field strength B increases, the magnetic torque exerted on the ST-MSCR also increases, resulting in a greater deflection angle θ. Furthermore, the relative error (see the Supplementary Materials) between the theoretical and measured values decreased as the magnetic field strength increased, with the smallest discrepancy of 2.29% observed at B=10 mT. In conclusion, the theoretical model proposed in this study provides an accurate estimation of the deflection angle of the ST-MSCR at relatively high magnetic field strengths (with a relative error of less than 5% when B ≥ 7 mT), demonstrating its practical applicability for design and optimization.

To verify the improvement of T-MSCR deflection performance by the tapered design, we first compared the deflection angles of the ST-MSCR (λ=20%) and different diameters of the SC-MSCR (D=0.6, 0.8, 1.0 mm, respectively, λ=20%) under magnetic field strengths ranging from 1 to 10 mT (φ=150°) using numerical simulations (Figure 4C). The results indicate that when the external magnetic field strength exceeds 3 mT, the ST-MSCR exhibits superior bending performance compared to the SC-MSCR with a distal diameter of 1.0 mm. This improvement is primarily due to the smaller distal diameter of the ST-MSCR, which, although it decreases the amount of magnetic particles and thus reduces the generated magnetic torque, also leads to a substantial reduction in bending stiffness. As the magnetic field strength increases, the influence of the reduced bending stiffness gradually becomes the dominant factor, thereby enhancing the deflection capability of the ST-MSCR.

To evaluate the optimization of magnetic particle distribution, numerical simulations were conducted to compare the deflection angles of the T-MSCR with those of SC-MSCRs of varying diameters (D=0.6,0.8,1.0 mm, λ=20%) under external magnetic field strengths ranging from 1 mT to 10 mT, with φ=150° (Figure 4C). The results indicated that the magnetic response bending ability of the T-MSCR was similar to that of the SC-MSCR with a diameter of 0.8 mm. This improvement is attributed to the optimization of magnetic particle distribution, which modulates the stiffness profile and mitigates the antagonistic effects between bending stiffness and magnetic torque in the T-MSCR. Finally, we experimentally compared the deflection angles of T-MSCR with ST-MSCR (λ=10, 15, 20%, respectively) under magnetic field strengths ranging from 1 to 10 mT (φ=180°) (Figure 4D), and measured the effect of λ on the deflection angle of ST-MSCR (Figure 4E). The results showed that under the same magnetic field strength, the T-MSCR exhibited the best bending performance, with a maximum deflection angle of 173.40° at 10 mT. Additionally, as λ increased, the deflection angle of the ST-MSCR first increased and then decreased, with the maximum deflection angle occurring at approximately λ=20%.

To evaluate the ability of the T-MSCR tip to assist in smoothly entering small vascular branches in narrow blood vessel paths, we designed four branched paths (Path1–Path4) with a diameter of 1.8 mm (turning angles: 30°, 40°, 65°, and 75°) based on the diameter of the posterior cerebral artery [45], as shown in Figure 4F. These increasing turning angles simulate progressively more challenging vessel entry scenarios. Here, the magnetic field strength range is defined as the difference between the upper and lower bounds of the magnetic field strength required for successful entry into the four branches. Subsequently, in a three-dimensional Helmholtz coil, we measured the range of magnetic field strengths required for both the T-MSCR and SC-MSCR (λ=20%, D=1 mm) to enter the four branches from the main branch (Figure 4G,H). The results indicated that within the designed paths, the T-MSCR had a wider magnetic field strength range than the SC-MSCR of the same size. This suggested that the T-MSCR had better magnetic field adaptability, making it easier to enter narrower vascular branches during magnetic navigation. This demonstrated that the tapered tip of the T-MSCR aids in its ability to enter small blood vessels.

Finally, due to the machining marks, the surface of the T-MSCR produced by injection molding was uneven. Additionally, the magnetic particles embedded in PDMS also presented potential exposure issues, posing a biosafety risk. Therefore, to improve the surface quality and biosafety, we coated T-MSCR with a layer of PDMS. Scanning electron microscope (SEM) images showed a significant improvement in the surface quality of the T-MSCR, as illustrated in Figure 4I.

### 3.5. Magnetic Steering and Selective Navigation of T-MSCR In Vitro

**Figure 5:**
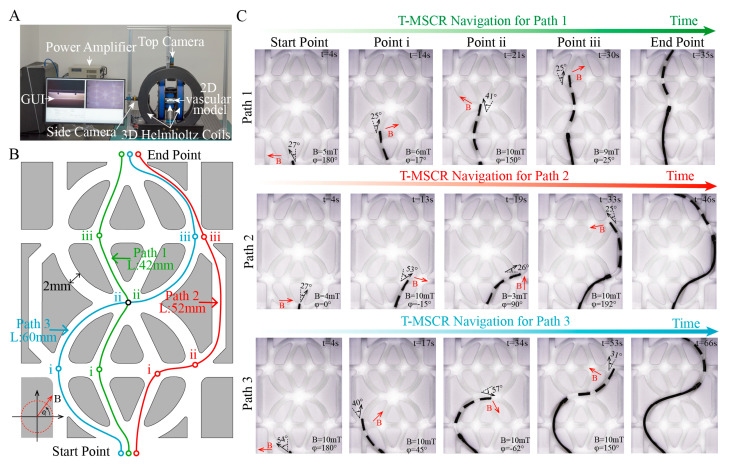
In vitro demonstration of T-MSCR magnetic steering and selective navigation. (**A**) Experimental setup including the three-dimensional Helmholtz coil magnetic navigation system, high-resolution camera, and two-dimensional vascular model. (**B**) Schematic of Path 1, Path 2, and Path 3 in the two-dimensional vascular model, where the starting and ending points are the same, but the lengths and turning angles vary. (**C**) The state of T-MSCR at each bifurcation point for the three paths. Red arrows indicate the direction of the applied magnetic field. This experiment was repeated three times to assess the time spent on navigation for each path.

To assess the magnetic steering capability and selective navigation performance of the T-MSCR in complex and narrow blood vessels, we conducted in vitro experiments using a two-dimensional vascular model that mimics the constricted and curved conditions of brain blood vessels. The T-MSCR was guided along various paths by adjusting the external magnetic field. The experimental setup consisted of a two-dimensional vascular model, a three-dimensional Helmholtz coil magnetic navigation system (MNS), and a high-resolution camera (Figure 5A). The vascular model was 3D printed from transparent photosensitive resin, with a channel width of 2 mm. Three distinct paths, each with the same start and end points, were designed, with Path 1, Path 2, and Path 3 having lengths of 42 mm, 52 mm, and 60 mm, respectively, as shown in Figure 5B.

**Figure 6:**
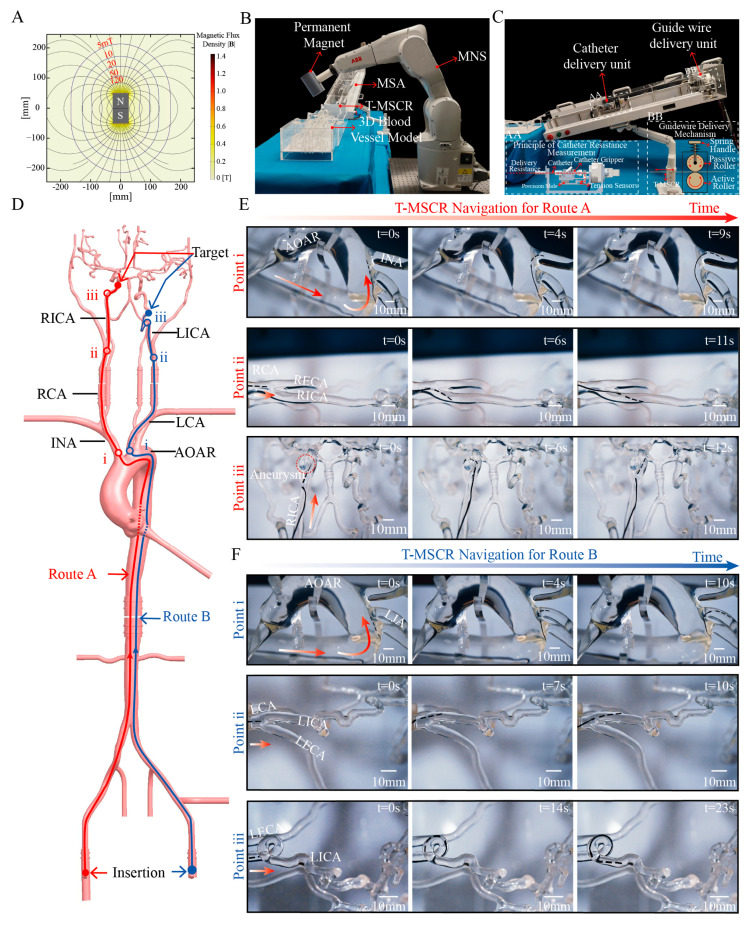
Demonstration of T-MSCR navigation in a human-scale vascular model in vitro. (**A**) The permanent magnet used in the magnetic navigation system generates an axisymmetric magnetic field distribution. (**B**) The designed MDGIS, where a 6-degree-of-freedom robotic arm controls the permanent magnet to form the MNS. (**C**) The distal part of the MSA in the MDGIS. (**D**) Schematic of two interventional routes in the human-scale vascular model: Route A starts at the RCFA and targets the aneurysm in the RICA, while Route B starts at the LCFA and targets the 180° bend in the LICA. (**E**) T-MSCR navigation in Route A, with three main turning points: 1. Turning from AOAR to INA, 2. Turning from RCA to RICA, and 3. Turning from RICA to the aneurysm. (**F**) T-MSCR navigation in Route B, with three main turning points: 1. Turning from AOAR to LCA, 2. Turning from LCA to LICA, and 3. Turning from LICA to the 360° bend. The experiment was repeated three times to evaluate the time spent at each turning point.

In the experiments, the MNS controlled the magnetic field direction at the turning points, ensuring it was perpendicular to the path direction, and applied a controlling torque by adjusting the magnetic field strength to guide the T-MSCR in selectively completing the path navigation. Meanwhile, the MSA (Figure 6C) was used to control the delivery of the T-MSCR. The T-MSCR was placed at the starting point of the vascular model, with the initial direction of the external magnetic field set perpendicular to the path direction. For all three paths, the T-MSCR started from the start point, selectively passed through their own predesigned bifurcation points i, ii, and iii, and finally reached the end point. As shown in Figure 5C, Path 1 had the shortest length and smallest turning angle, and the entire process took 35 ± 2.8 s. Path 2 had only one bifurcation point (i) with a relatively large turning angle, and the entire process took 46 ± 6.3 s. Path 3 had both larger turning angles and the longest path length, so the entire navigation process took the longest time, 66 ± 8.5 s. The experimental results showed that the T-MSCR had no major difficulties or unexpected movements that occurred during the entire process (Video S2). These in vitro demonstrations clearly show that the tapered tip of the T-MSCR, owing to its excellent bending ability, can flexibly guide the T-MSCR to navigate through narrow bifurcation points. The T-MSCR was not operated by a professional interventionalist, and its excellent magnetic steering and selective navigation capabilities in the two-dimensional vascular model indicate its ability to adapt to complex vascular environments.

### 3.6. In Vitro Navigation Within Human Vascular Model

To validate the navigation performance of T-MSCR in a real-scale vascular model, we designed and constructed an MDGIS experimental setup. In MDGIS, the MNS section uses a 6-degree-of-freedom robotic arm (IRB1200-7/0.7; ABB, Zurich, Sweden) to control a permanent magnet, as shown in Figure 6B. The robotic arm had a repeat positioning accuracy of 0.02 mm and was used to control the position of a cylindrical axial magnet (N52 grade, 5 cm in diameter, 9 cm in thickness). This magnet generates a non-uniform magnetic field of 10 mT at a distance of 17 cm, and the field direction and position can be dynamically adjusted via the robotic arm to ensure stable navigation of the T-MSCR in complex paths. The axially symmetric magnetic field generated by the permanent magnet is shown in Figure 6A. The MSA section of the MDGIS is shown in Figure 6C, which simulates the interventional physician’s operations of guidewire and catheter delivery, retraction, rotation, and tactile feedback during the delivery process to enable the remote delivery of the guidewire and catheter.

The vascular model used in the experiment was based on real human anatomical data and was made of transparent silicone using 3D printing technology. It was used to evaluate the ability of the T-MSCR to navigate cerebrovascular lesions (Figure S10). Because of the significant friction between the T-MSCR and the inner wall of the silicone model, whereas the friction coefficient between vascular interventional devices and real blood vessels is typically very low (<0.046), we mixed water and sodium fatty alcohol sulfate in a 20:1 mass ratio to prepare a solution. To better simulate the real vascular environment, the mixed solution was injected into the three-dimensional vascular model to reproduce the actual friction conditions between the guidewire and the vessel [13,46].

The entire vascular model includes a complete path from the common femoral artery (CFA) to the intracranial arteries, specifically the CFA, aortic arch (AOAR), carotid artery, and intracranial arteries. A simulated cerebral aneurysm was placed in the right internal carotid artery (RICA). Additionally, multiple complex bends were designed in the siphon segment of the left internal carotid artery (LICA), including two 180° and two 360° bends, to simulate the navigation challenges in the complex intracranial vasculature (Figure 6D).

For difficult-to-reach intracranial arteries, two vascular navigation routes were designed, as illustrated in Figure 6D. Route A contains the right common femoral artery (RCFA), AOAR, innominate artery (INA), right carotid artery (RCA), and right internal carotid artery (RICA), leading to the target aneurysm. Key turning points for Route A include the transitions from the AOAR to the INA, from the RCA to the RICA, and from the RICA to the aneurysm. Route B contains the left common femoral artery (LCFA), AOAR, left carotid artery (LCA), and LICA, followed by a 360° bend and a 180° bend within the LICA. Key turning points include the transitions from the AOAR to the LCA, from the LCA to the LICA, and the passage through the 360° bend to reach the 180° bend.

Navigation experiments were conducted for both routes. In each experiment, we coordinated the turning and delivery of the T-MSCR by synchronously controlling the position of the external permanent magnet in the MNS system while operating the master end of the MSA. Every time the T-MSCR reached a turning point, we set t = 0 s. Figure 6E and Video S3 illustrate the complete navigation procedure for Route A. First, the T-MSCR passed the first turning point (i) and reached the INA in 9 ± 3 s. It then continued through the second turning point (ii), reaching the RICA in 11 ± 3.4 s. Subsequently, it reached the aneurysm, spending 6 ± 1.3 s entering the aneurysm. Finally, the aneurysm was left in 12 ± 3 s. Figure 6F and Video S4 illustrate the complete navigation procedure for Route B. First, the T-MSCR passed the first turning point (i) and reached the LCA in 10 ± 3.6 s. It then passed through the second turning point (ii) and reached the LICA in 10 ± 2.3 s. Finally, it navigated through the 360° bend and reached the 180° bend in 23 ± 3.8 s. These results demonstrate that the T-MSCR has excellent flexibility and navigation capability in restricted vascular environments, efficiently handling the navigation demands of continuous curves and multi-branch paths.

### 3.7. Biosafety of T-MSCR

To evaluate the biosafety of T-MSCR for interventional therapy, we co-cultured T-MSCR samples with human umbilical vein endothelial cells (HUVECs). In the experiment, 2 mm long segments of T-MSCR samples were cut from the same position as the test materials, and two experimental conditions were set: (1) Control group (Ctrl), cultured using HUVEC-specific medium (with fetal bovine serum) without T-MSCR for a duration of three days; (2) Experimental group (T-MSCR), cultured using HUVEC-specific medium (with fetal bovine serum) containing T-MSCR samples for a duration of three days.

**Figure 7:**
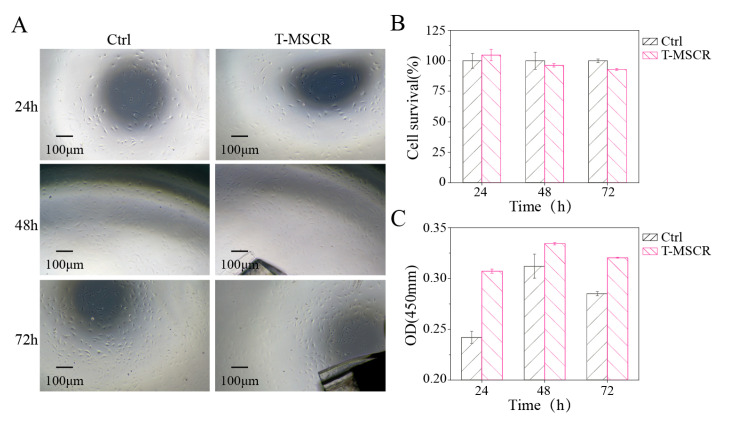
Biosafety assessment of T-MSCR. (**A**) Bright-field microscope images of HUVEC cell proliferation in the T-MSCR group and the Ctrl group. (**B**) Cell viability was calculated from absorbance values. (**C**) Absorbance values at a wavelength of 450 nm.

To assess the morphology distribution and survival rate of HUVEC cells, the cells were observed using an inverted fluorescence microscope at 24, 48, and 72 h of culture (Figure 7A). After that, CCK-8 reagent was added for a 2-h incubation. Following incubation, 100 μL of supernatant was transferred to a 96-well plate. The absorbance at 450 nm was measured using a microplate reader (Figure 7C), and cell viability was calculated based on the absorbance values (Figure 7B). The results showed that the T-MSCR samples had no significant toxicity to HUVEC cells. At the end of the culture (72 h), both the experimental group (T-MSCR) and the control group (Ctrl) showed good cell proliferation ability, with cell viability of 96.73% in the experimental group, which was not significantly different from that of the control group (100.00%). Moreover, fluorescence microscope observations showed that the HUVEC cells in the experimental group had intact morphology, with no obvious morphological abnormalities or distribution disorders. Therefore, the designed T-MSCR exhibits good biosafety.

## 4. Discussion

This study proposes a T-MSCR design and optimizes the distribution of the magnetic particle based on the DGWO and Euler–Bernoulli beam theory with the aim of enhancing the deflection capability. This can help the T-MSCR navigate through narrow blood vessels. We fabricated three ST-MSCRs (λ=0, 20, 40%, respectively) using mold injection. Then, based on the optimization results, different segments were cut and spliced together to form a multi-segmented T-MSCR. To improve the surface quality of the T-MSCR and eliminate potential safety risks caused by the exposure of NdFeB particles, we uniformly coated the T-MSCR surface with a layer of PDMS. After curing, the T-MSCR was subjected to axial magnetization using a pulsed magnetizer. Finally, the fabricated T-MSCR was integrated with the distal end of a commercial guidewire, enabling its application in vascular navigation procedures.

We demonstrated the optimization process of the deflection capability of the T-MSCR based on the DGWO and compared the proposed robot deflection theoretical model with the actual end deflection angles. This comparison validated the predictive accuracy of the theoretical model regarding the effects of the magnetic field strength on the deflection angle. In addition, we experimentally verified the T-MSCR and compared its performance with those of SC-MSCR and ST-MSCR, confirming that T-MSCR of the same size has better deflection and guidance capabilities. Furthermore, in the two-dimensional restricted channel model, the path selection abilities of the T-MSCR were demonstrated. In the three-dimensional human vascular model, its navigation capability was illustrated. Finally, considering the clinical applications of T-MSCR, its biosafety is crucial for future promotion. To this end, we evaluated the biosafety of T-MSCR, and the results showed that T-MSCR was non-toxic to HUVEC cells and exhibited good biocompatibility.

Several key challenges must be addressed to bring T-MSCR to practical clinical applications. First, the current mold-casting method limits the flexibility in structural design and magnetization patterns. Recent advancements in magnetic field-assisted stereolithography offer the potential for the three-dimensional magnetic programming of complex soft robots, which could enhance the structural complexity, flexibility, and molding quality [47]. Additionally, the T-MSCR currently lacks force feedback functionality. While force sensors in the MSA system measure friction between the guidewire and vessel wall, they do not precisely capture the resistance at the guidewire tip. As vascular perforation risk is primarily due to excessive tip resistance, future developments will focus on incorporating force feedback technology at the tip to provide real-time tactile information and improve the safety of interventional procedures. Moreover, currently, experimental conditions are limited to static environments, and future studies will employ dynamic vascular models to investigate the effects of blood flow and vessel wall motion on T-MSCR performance. Finally, the PDMS surface of the T-MSCR, characterized by low modulus and high elasticity, deforms upon contact with the vessel wall, resulting in an increased real contact area and elevated friction. This friction may impede advancement and induce bending under axial load, thereby compromising delivery. Additionally, the magnetic field gradients generated by the MNS system can cause unintended bending, further impairing navigation. To address these issues, a hydrophilic coating will be applied to reduce friction, and a stiff nickel-titanium alloy wire will be embedded in the core to counteract bending induced by magnetic field gradients.

## Data Availability

The original contributions presented in the study are included in the article; further inquiries can be directed to the corresponding author.

## Supplementary Materials

The following supporting information can be downloaded at: https://www.mdpi.com/article/10.3390/mi16060701/s1, Note S1: Theoretical modeling of T-MSCR deformation; Note S2: Segmented linear fitting; Note S3: GA-based Optimization Process for T-MSCR; Note S4: DPSO-based Optimization Process for T-MSCR; Note S5: Relative error calculation; Note S6: T-MSCR Fabrication Process; Note S7: Cell viability calculation; Figure S1: Segmented linear fitting diagram; Figure S2: Schematic of the segmented linear fitting process; Figure S3: GA optimization workflow for T-MSCR; Figure S4: DPSO optimization workflow for T-MSCR; Figure S5: Density of T-MSCR composites varying with magnetic particle volume ratio; Figure S6: Iteration convergence of T-MSCR deflection angle under different external magnetic field angles φ; Figure S7: Geometric representation of the elastic deformation model of the T-MSCR under an external magnetic field; Figure S8: Schematic of the delivery mechanism for MSA; Figure S9: Real Object Image of the MSA; Figure S10: Human vascular model; Video S1: Tensile Test; Video S2: T-MSCR Magnetic Steering and Selective Navigation Capabilities in a dimensional Vascular Model; Video S3: Navigation of right internal carotid artery aneurysm by T-MSCR on Route A in a three-dimensional vascular model; Video S4: Navigation of the 180-degree curvature pattern of the left internal carotid artery by MSCR on Route B in a three-dimensional vascular model.

## Abbreviations

The following abbreviations are used in this manuscript:

MSCR	Magnetic soft continuum robot
T-MSCR	Tapered MSCR
GWO	Gray Wolf Optimizer
DGWO	Discrete GWO
MDGIS	Magnetic-driven guidewire interventional systems
MNS	Magnetic navigation system
MSA	Master-slave advancer
SC-MSCR	Single-concentration cylindrical MSCR
ST-MSCR	Single-concentration tapered MSCR
PDMS	Polydimethylsiloxane
HUVEC	Human umbilical vein endothelial cells
GA	Genetic Algorithm
DPSO	Discrete Particle Swarm Optimization
SEM	Scanning electron microscope
CFA	Common femoral artery
AOAR	Aortic arch
RICA	Right internal carotid artery
LICA	Left internal carotid artery
RCFA	Right common femoral artery
INA	Innominate artery
RCA	Right carotid artery
LCFA	Left common femoral artery
LCA	Left carotid artery
