# Supplementary material for: Design and Optimization of a Tapered Magnetic Soft Continuum Robot for Enhanced Navigation in Cerebral Vasculature
Source: Micromachines (Basel). 2025 Jun 12;16(6):701. doi: 10.3390/mi16060701 (PMC12195137; doi:10.3390/mi16060701)
Supplement: Supplementary file 1 [file micromachines-16-00701-s001.zip › supplementary materials/SI appendix.pdf]

Supporting Information (SI) for

Design and Optimization of a Tapered Magnetic Soft Continuum Robot for Enhanced Navigation in Cerebral Vasculature

Jiahang Wang †, Yuhang Liu †, Xiwen Lu, Yunlong Zhu and Chenyao Bai *

* Correspondence: baichenyao@fudan.edu.cn

This PDF file includes:

Supporting Method Notes S1-S7

Figures S1 to S10

Movie Legends S1 to S4

Supporting Methods

Note S1: Theoretical modeling of T-MSCR deformation

The bending and deformation of the ST-MSCR are induced by the interaction between the actuation magnetic field and the hard magnetic particles dispersed within the polymer matrix. In a uniform magnetic field, this interaction manifests as a distributed magnetic torque. Given that the strength of the actuation magnetic field is substantially lower than the coercive force of the hard magnetic particles, it can be assumed that the magnetization intensity of each part of the ST-MSCR remains constant during deformation. In a stable state, the deformation of the ST-MSCR results from the equilibrium between the magnetic torque applied by the driving magnetic field and the bending moment within the elastomer. First, the proximal diameter, distal diameter, and length of the ST-MSCR are defined as d_B, d_A, and L, respectively. The diameter ratio is defined as $\alpha = d_B/d_A$ ($\alpha > 1$). Due to the slender nature of the ST-MSCR ($L \gg d_B > d_A$), we employed the Euler-Bernoulli beam theory to describe the large deformation of the ST-MSCR under a magnetic field. This theory posits that during deformation, an axial neutral line whose length remains unchanged exists, and the cross-sections at various points remain perpendicular to this axial neutral line. Consequently, the magnetic torque-bending moment equilibrium equation can be established as follows:

$$EI\,\kappa\,(s) = \int_V (\boldsymbol{M} \times \boldsymbol{B})dV \qquad (S1)$$

Where EI denotes the bending stiffness of the ST-MSCR, and $\kappa(s) = d\theta/ds$ represents the curvature of the axial neutral line at the arc length coordinate s. The symbol $\times$ indicates the vector cross product. Eq.1-1 describes the equilibrium state where the bending moment at any point on the ST-MSCR is equal to the sum of the magnetic torques acting from that point to the free end. Considering the geometric characteristics of the elephant trunk-inspired ST-MSCR, we can further refine Eq.S1 into the following form:

$$EI\frac{d\theta}{ds} = \frac{\pi d_A^2}{4L^2}\int_{s_0}^{L} MB\,sin(\varphi - \theta(s))Q(s)ds \qquad (S2)$$

Where, $\theta(s)$ represents the angle between the neutral line tangent at the arc length coordinate s and the initial direction of the ST-MSCR. $Q(s) = [(1-\alpha)x + \alpha L]^2$ donates the volume correction term. Given specific initial value (e.g.,$\theta(0) = 0$ at the fixed boundary), solving Eq.S2 yields the tangent angle $\theta(s)$ at various positions along the neutral line of the ST-MSCR, thus determining the deflection angle of the ST-MSCR $\theta(s = L)$.

For T-MSCR with multiple magnetic particle volume ratios, Eq.S2 can be solved using the finite difference method. Initially, the entire elastomer is discretized into N equal-length

elements, where the arc length differential element can be approximated by a segment of a straight line (i.e., $ds \approx \Delta s = L/N$). The curvature at any point on the neutral line can be approximated as $\kappa(s) = d\theta/ds \approx \theta_i - \theta_{i-1}$, where θ_i denotes the angle between the i_{th} element and the reference direction. By adjusting the value of N, we can ensure that the magnetic particle volume ratio λ is consistent within each element. Therefore, Equation S2 can be further rewritten in the following finite difference form:

$$E_i I_i \frac{\theta_i - \theta_{i-1}}{\Delta s} = \frac{\pi d_A^2}{4L^2} \sum_{q=i}^{N} M_q B \, sin(\varphi - \theta_q) Q_q \Delta s \, , i = 1,2,3, \dots, N \qquad (S3)$$

It can be observed that Equation S3 forms a N-dimensional system of nonlinear equations, involving N unknowns $[\theta_1, \theta_2, \theta_3, \dots, \theta_N]$. By incorporating the initial value $\theta_0 = 0$, the deflection angle θ_N and the coordinates of any point on the elastomer can be determined (Equation S4).

$$\begin{cases} x = \sum_{q=1}^{N} cos\, \theta_q \, \Delta s \\ y = \sum_{q=1}^{N} sin\, \theta_q \, \Delta s \end{cases} \qquad (S4)$$

Note S2:Segmented linear fitting

To facilitate sample preparation, we fitted the distribution of the magnetic particle volume ratio of T-MSCR using the red line in Figure S1. Specifically, inspired by image filtering algorithms, we set up a sliding window with a length of 10 voxels. Aligning the starting position of this window with the first voxel of the optimized magnetization pattern, we recorded the most frequently occurring magnetic particle volume ratio λ within the window and set the volume ratio of magnetic particles at all other positions within the window to this value. The window was then shifted backward by 10 voxels. This process was repeated until the window reached the boundary of the magnetization pattern, as illustrated in Figure S2.

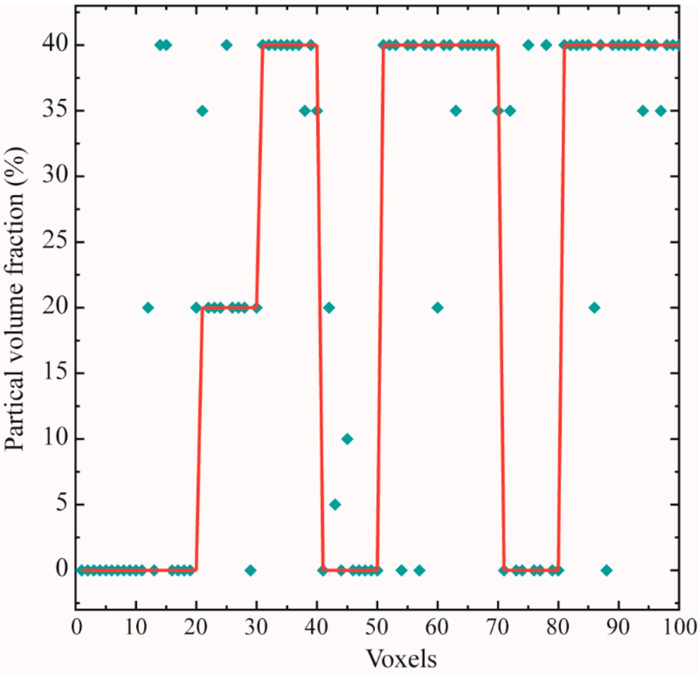

Figure S1. Segmented linear fitting diagram

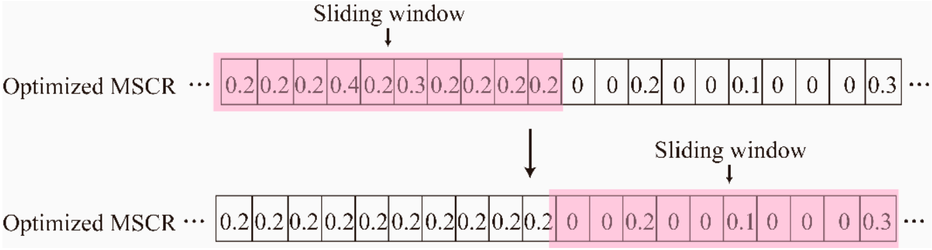

Figure S2. Schematic of the segmented linear fitting process

Note S3: GA-based Optimization Process for T-MSCR

The overall workflow of GA is illustrated in Figure S3. Specifically, during the initialization phase, the magnetized distal section of the T-MSCR was discretized into 100 equal-length voxels. Each voxel was assigned a randomly selected magnetic particle volume ratio λ from the discrete set $\{0, 0.1, 0.15, 0.2, 0.25, 0.3, 0.35, 0.4\}$. An initial population $\mathbf{X^0}$ comprising 50 individuals was then generated. The external magnetic field was set to have a magnetic flux density of $B = 10mT$ and a direction angle of $\varphi = 150°$.

During the iteration process, the deflection angle $\theta(x_i^{iter})$ of each individual was calculated using the finite difference model. A stochastic universal sampling strategy was then employed to select 50 individuals exhibiting the largest deflection angles. The next generation was produced from these selected T-MSCRs using a genetic strategy consisting of 10% elitism, 80% crossover, and 10% mutation. Specifically, elitism preserved the top ten T-MSCRs with the highest deflection angles, which were directly passed to the next generation without modification. For crossover, 80 MSCRs were randomly selected from the remaining 90

individuals and paired to exchange portions of their voxel configurations. For mutation, the remaining 10 individuals underwent probabilistic voxel alterations to generate new offspring. This iterative process—comprising deflection angle calculation, stochastic universal sampling, and genetic operations—was repeated until the difference between the maximum deflection angle and the average deflection angle across the population fell below the predefined threshold (10^{-3}).

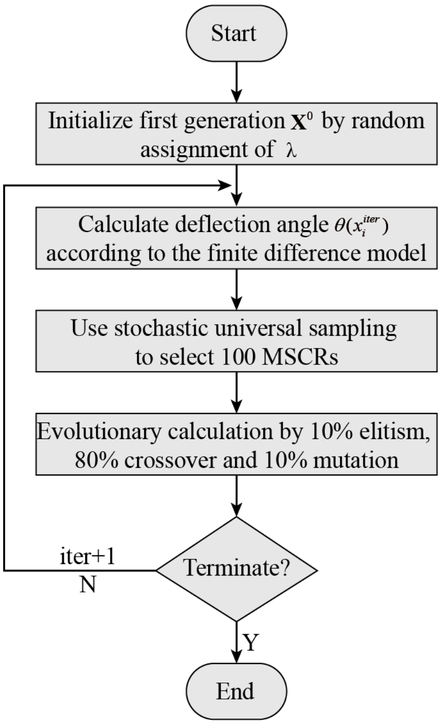

Figure S3. GA optimization workflow for T-MSCR

Note S4: DPSO-based Optimization Process for T-MSCR

The overall workflow of DPSO is illustrated in Figure S4. In the initialization phase of the DPSO for optimizing the magnetic particle distribution of the T-MSCR, the magnetized distal segment of the MSCR was discretized into 100 equal-length voxels. Each voxel was assigned a randomly selected magnetic particle volume ratio λ from the discrete set $\{0, 0.1, 0.15, 0.2, 0.25, 0.3, 0.35, 0.4\}$. An initial particle swarm $\mathbf{X^0}$ comprising 100 particles was then generated. Additionally, the initial velocity v_i^0 of each particle was randomly initialized from the discrete set $\{-1, 0, 1\}$. The external magnetic field was configured with a magnetic flux density of $B = 10mT$ and an orientation angle of $\varphi = 150°$.

In the iteration process, the deflection angle $\theta(x_i^{iter})$ for each particle in the current swarm was first computed using the finite difference model. Subsequently, the velocity v_i^{iter+1} and position x_i^{iter+1} of each particle i were updated according to Equations S5–S7.

$$z_i^{iter+1} = \omega v_i^{iter} + c_1 r_1 \cdot \left(pbest - x_i^{iter}\right) + c_2 r_2 \cdot \left(gbest - x_i^{iter}\right) \qquad (S5)$$

$$v_i^{iter+1} = \begin{cases} 1, \ z_i^{iter+1} > v_{thre} \\ 0, \quad othrewise \\ -1, z_i^{iter+1} < -v_{thre} \end{cases} \tag{S6}$$

$$x_i^{iter+1} = x_i^{iter} + v_i^{iter+1} \cdot step \tag{S7}$$

Where x_i^{iter} denotes the current position of particle i; ω is the inertia weight, set to 0.9; c_1 and c_2 are the cognitive and social learning factors, respectively, set to 2.5 and 1.5; r_1 and r_2 are 10-dimensional random vectors, with each element uniformly distributed in the range 0~1; pbest and gbest represent the historical individual best solution of the current particle and the global best solution of the entire swarm during the iteration process, respectively; z_i^{iter+1} denotes the temporary velocity; v_{thre} is the velocity threshold, set to 0.2; $step$ refers to the increment in magnetic particle volume ratio between adjacent values in the λ set ($step$ = 0.05 in this study).

This iterative process involves computing the deflection angle for each particle, updating particle velocities and positions accordingly, and generating the next-generation swarm. Once the maximum number of iterations is reached, the particle associated with gbest is regarded as the optimal solution for the magnetic particle distribution.

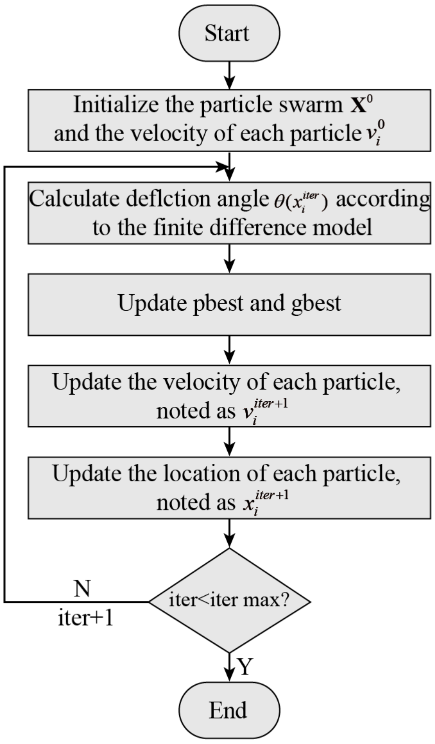

Figure S4. DPSO optimization workflow for T-MSCR

Note S5: Relative error calculation

The relative error between theorical and experimental values of T-MSCR is calculated as follows:

$$\epsilon_r = \frac{|\theta_t - \theta_e|}{\theta_t} \quad (S8)$$

Where θ_t represents the theoretical deflection angle of the T-MSCR obtained using the finite difference model; θ_e represents the experimentally measured deflection angle of the T-MSCR.

Note S6: T-MSCR Fabrication Process

First, based on the optimized volume ratio of magnetic particles and the PDMS-to-curing agent weight ratio (19:1) determined in the T-MSCR design, the required masses of PDMS, curing agent, and magnetic particles were calculated. These components were sequentially weighed and added into a beaker, followed by thorough mixing using a mechanical stirrer at 1000 rpm for 2 minutes. The resulting mixture was then placed in a vacuum drying oven to remove air bubbles.

After degassing, the magnetic composite mixture was injected into a tapered mold (Figure 4A(i)) and cured in a vacuum drying oven at 100 °C for 2 hours. Upon curing, the composite was demolded to obtain ST-MSCRs ($\lambda = 0{,}20{,}40\%$). These ST-MSCRs were then trimmed into sub-sections according to the optimized T-MSCR design.

The sub-segments were assembled in a tapered mold for alignment and bonded using PDMS (Figure 4A(v)). The assembled structure was subsequently cured again in a vacuum drying oven at 100 °C for 2 hours. Afterward, a thin layer of PDMS was coated on the T-MSCR surface and thermally cured under the same conditions for another 2 hours.

Finally, axial magnetization was applied to the T-MSCR using a pulse magnetizer (Figure 4A(vi)). The magnetized T-MSCR was then bonded to a commercial guidewire using adhesive, and the connection area was reinforced with heat-shrink tubing.

Note S7:Cell viability calculation

The cell viability is calculated as follows:

$$Cell\ viability(\%) = \frac{As - Ab}{Ac - Ab} \times 100\% \quad (S9)$$

Where As is the absorbance of the T-MSCR group wells (containing cells, culture medium, CCK-8, and T-MSCR); Ab is the absorbance of the blank wells (containing culture medium and CCK-8); Ac is the absorbance of the Ctrl group wells (containing cells, culture medium, and CCK-8).

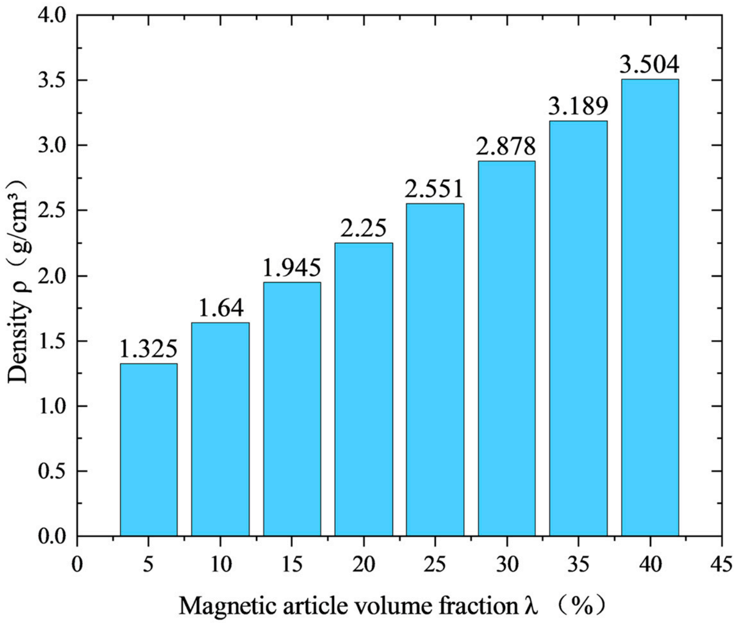

Figure S5. Density of T-MSCR composites varying with magnetic particle volume fraction

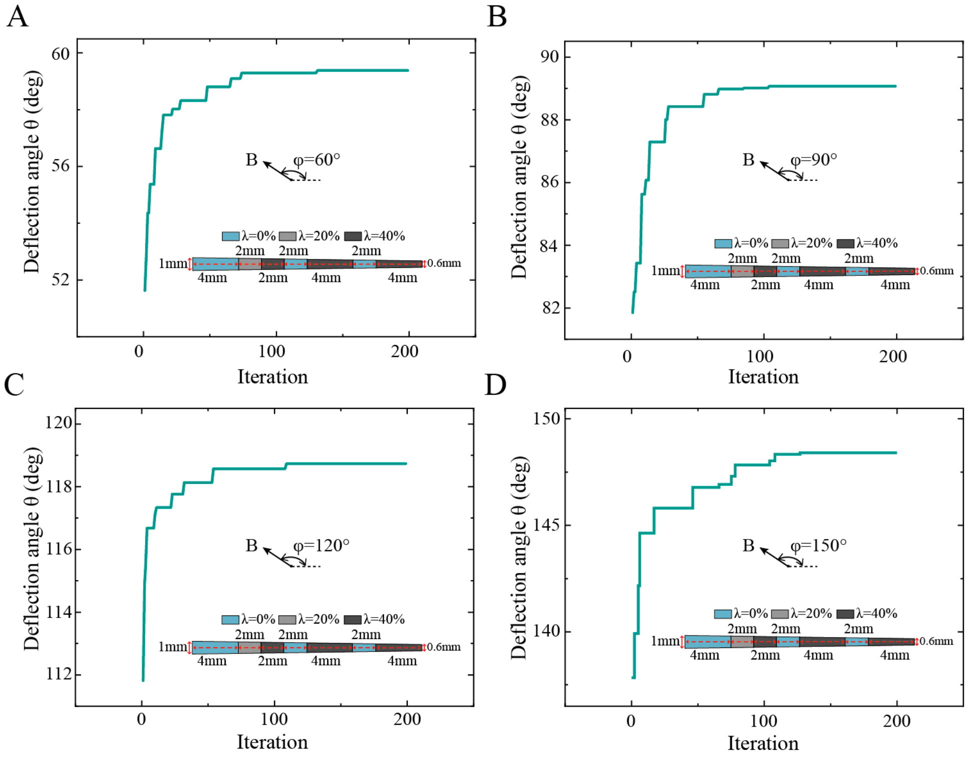

Figure S6. Iteration convergence of T-MSCR deflection angle under different external magnetic field angles φ. A) $\varphi = 60°$, B) $\varphi = 90°$, C) $\varphi = 120°$, D) $\varphi = 150°$

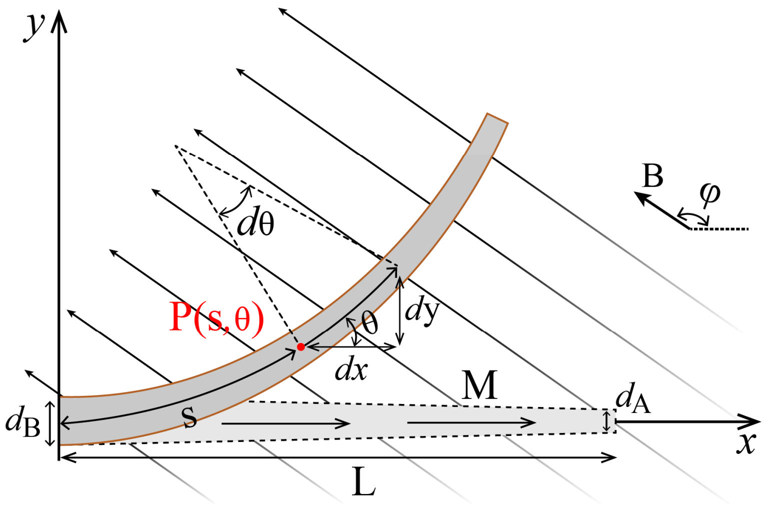

Figure S7. Geometric representation of the elastic deformation model of the T-MSCR under an external magnetic field. The deformed is described by a parameterized spatial curve $\theta = \theta(s)$, known as the elastic line. Here, s represents the arc length from the origin to any point P on the elastic line, and θ denotes the tangential angle at the spatial point $P(s, \theta)$.

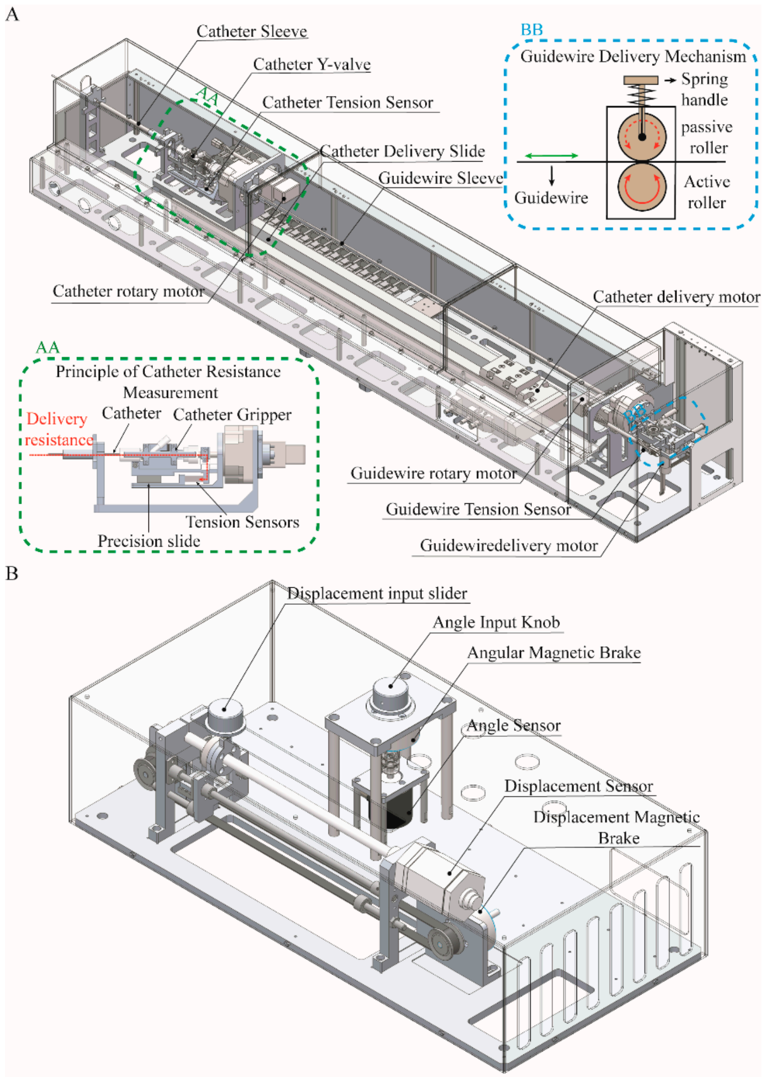

Figure S8. Schematic of the delivery mechanism for MSA. A) Slave of MSA. B) Master of MSA.

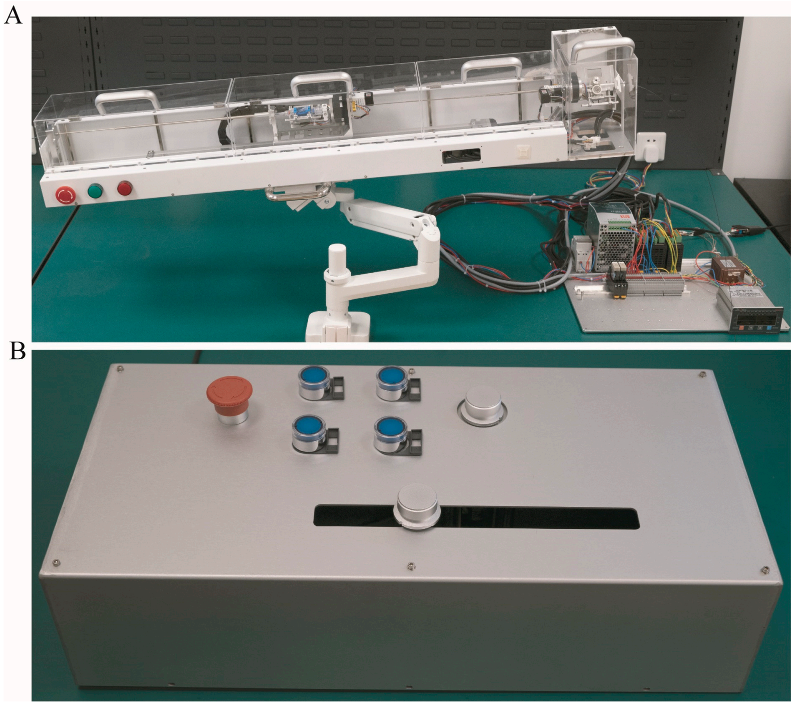

Figure S9. Real Object Image of the Master-Slave Advancer (MSA)**.** A) Slave of MSA. B) Master of MSA.

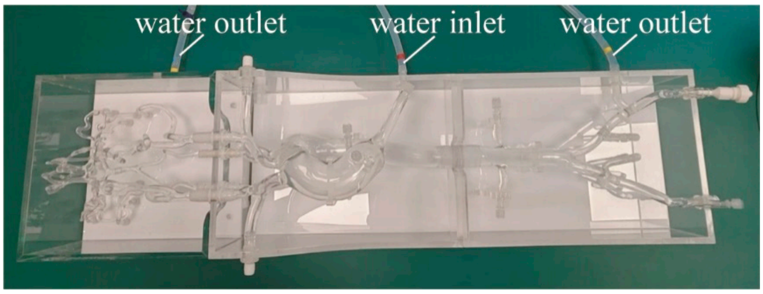

Figure S10. Human vascular model.

Movie legends

Movie S1. Tensile Test

Movie S2. T-MSCR Magnetic Steering and Selective Navigation Capabilities in a two-dimensional Vascular Model

Movie S3. Navigation of right internal carotid artery aneurysm by T-MSCR on Route A in a three-dimensional vascular model

Movie S4. Navigation of the 180-degree curvature pattern of the left internal carotid artery by MSCR on Route B in a three-dimensional vascular model
